# Feasibility of Laser-Induced Breakdown Spectroscopy and Hyperspectral Imaging for Rapid Detection of Thiophanate-Methyl Residue on Mulberry Fruit

**DOI:** 10.3390/ijms20082017

**Published:** 2019-04-24

**Authors:** Di Wu, Liuwei Meng, Liang Yang, Jingyu Wang, Xiaping Fu, Xiaoqiang Du, Shaojia Li, Yong He, Lingxia Huang

**Affiliations:** 1 College of Agriculture & Biotechnology/Zhejiang Provincial Key Laboratory of Horticultural Plant Integrative Biology/The State Agriculture Ministry Laboratory of Horticultural Plant Growth, Development and Quality Improvement, Zhejiang University, Zijingang Campus, Hangzhou 310058, China; di_wu@zju.edu.cn (D.W.); 11216044@zju.edu.cn (S.L.); 2 College of Animal Sciences, Zhejiang University, Hangzhou 310058, China; 21517069@zju.edu.cn (L.M.); lyoung1101@163.com (L.Y.); Jingyu529@zju.edu.cn (J.W.); 3 Faculty of Mechanical Engineering & Automation, Zhejiang Sci-Tech University, Hangzhou 310018, China; fuxp@zstu.edu.cn (X.F.); xqiangdu@zstu.edu.cn (X.D.); 4 Key Laboratory of Transplanting Equipment and Technology of Zhejiang Province, Hangzhou 310018, China; 5 College of Biosystems Engineering and Food Science, Zhejiang University, Zijingang Campus, Hangzhou 310058, China; yhe@zju.edu.cn; 6 South Taihu Agricultural Technology Extension Center in Huzhou, Zhejiang University, Huzhou 313000, China

**Keywords:** mulberry, laser-induced breakdown spectroscopy, hyperspectral imaging, pesticide residue, chemometrics, variable selection

## Abstract

An effective and rapid way to detect thiophanate-methyl residue on mulberry fruit is important for providing consumers with quality and safe of mulberry fruit. Chemical methods are complex, time-consuming, and costly, and can result in sample contamination. Rapid detection of thiophanate-methyl residue on mulberry fruit was studied using laser-induced breakdown spectroscopy (LIBS) and hyperspectral imaging (HSI) techniques. Principal component analysis (PCA) and partial least square regression (PLSR) were used to qualitatively and quantitatively analyze the data obtained by using LIBS and HSI on mulberry fruit samples with different thiophanate-methyl residues. The competitive adaptive reweighted sampling algorithm was used to select optimal variables. The results of model calibration were compared. The best result was given by the PLSR model that used the optimal preprocessed LIBS–HSI variables, with a correlation coefficient of 0.921 for the prediction set. The results of this research confirmed the feasibility of using LIBS and HSI for the rapid detection of thiophanate-methyl residue on mulberry fruit.

## 1. Introduction

The mulberry is a fruit offering health benefits because it is rich in nutrients and bioactive substances such as antioxidants and alkaloids [1]. Mulberries are susceptible to pests and diseases, resulting in the decrease of both production and quality. Large quantities of thiophanate-methyl are sprayed on mulberries to prevent pests and diseases. As a typical representative of berry, mulberry has the characteristics of thin skin and thick flesh, which will be more likely to accumulate excess pesticide on fruit and cause the pesticide residue to exceed the standard. In the management of an orchard, the use of thiophanate-methyl usually leads to the problem of fungicide residue on the fruit. Therefore, thiophanate residue is an actual problem for many fruits like mulberry. Pesticide residues are a serious threat to fruit safety and the environment, and in general, they are harmful to human health [2]. Thus, it is important to establish a quick and effective method of detecting thiophanate-methyl residue on mulberries in order to minimize the effects of the fungicide residue on human health.

Pesticide residues are commonly detected using analytical methods, such as gas chromatography (GC) [3], gas chromatography–mass spectrometry (GC-MS) [4], high-performance liquid chromatography (HPLC) [5], ultra-high-performance liquid chromatography–mass spectrometry (UPLC-MS) [6], capillary electrophoresis [7], immunoassay [8,9], and biosensor applications [10]. However, these methods are complex, time-consuming, and costly, and may result in sample contamination. Laser-induced breakdown spectroscopy (LIBS) is a novel analytic technique that can rapidly analyze samples via the spectroscopic analysis of laser ablation products [11,12]. The component elements of the observed samples can be qualitatively and quantitatively analyzed according to the position and intensity of the emission spectra. As a fast and effective element detection technology, LIBS has been widely used in many fields, such as monitoring of the heavy metal pollution in environmental soil [13], detection of elements in steel [14] and coal [15], and detection of nutrients and heavy metals in rice [16] and vegetable [17]. Additionally, LIBS has been used for detecting pesticide residue, including detecting pesticides in foods such as spinach powder and rice pellets [18], apples and pears [19,20], and tissue fats and rendering oils [21]. Hyperspectral imaging (HSI) is another promising technique that combines spectroscopy with imaging [22]. HSI simultaneously provides abundant spectral information and spatial images of the surface of the sample [23,24,25]. Accurate noiseless spectral information of a sample can be extracted from a hyperspectral image. HSI has been widely used to measure the quality of agricultural products such as fruit, vegetables, meat, fish, and crops [26]. There have been studies that used visible–near infrared HSI techniques to detect pesticides on microalgae [27], chlorella pyrenoidosa [28], and fruit peels [29]. These studies indicate that LIBS and HSI both provide ways to detect pesticides. However, their results need to be improved, and other detection techniques are needed in combination with HSI to further increase the accuracy of pesticide detection.

LIBS identifies the spectra of elements, whereas HSI is commonly used to identify the absorption peaks of chemical bonds and functional groups in organic matter in the visible and near-infrared bands. We suggest that the two together make it possible to quickly and simply detect fungicide residue on mulberries by identifying both molecular bond vibrations and elements [30]. To our knowledge, there are few studies that have investigated the rapid detection of pesticide residues on fruit or other agricultural products by combining LIBS and HSI. The main goal of this work is to assess the feasibility of using LIBS and HSI techniques to detect thiophanate-methyl residue on mulberries and the rapid detection of thiophanate residues on mulberry. A novel approach of combining LIBS and HSI techniques is evaluated to improve the ability to predict and detect pesticide residue.

## 2. Results

### 2.1. Features of LIBS and HSI Spectra of Mulberry Fruit Contaminated by Thiophanate-Methyl

**Figure 1:**
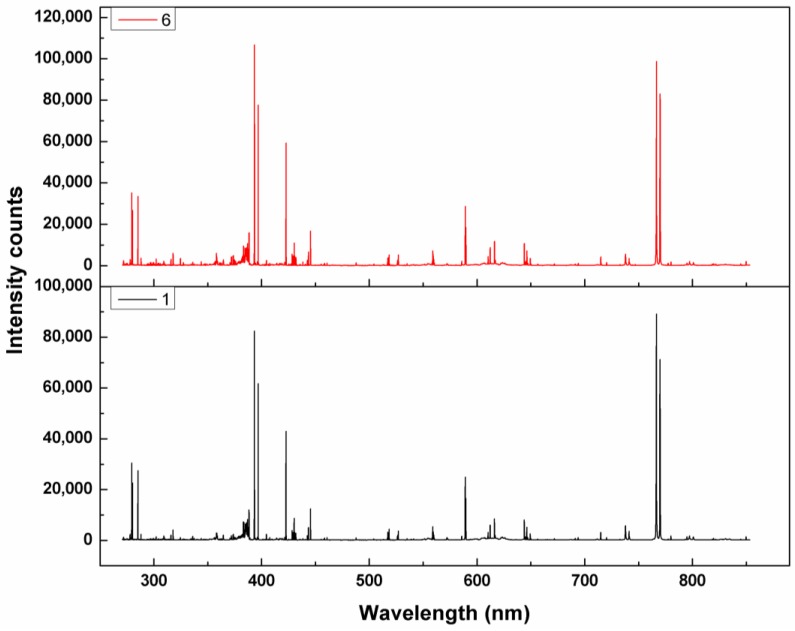
Typical LIBS profiles of the control group and pesticide residue group of mulberry fruit with two concentrations of the pesticide solution (0 g mL^−1^ and 0.0001 g mL^−1^).

LIBS data for mulberry fruit were obtained with differing thiophanate-methyl residues. Figure 1 shows two typical LIBS spectra from the control group and pesticide residue group in the region 270–850 nm. The spectral profiles are similar to each other. No obvious difference in spectral lines and intensities can be found by direct visual analysis of the spectra. Some of the emission peaks (from Fe, Cs, Th, and Sr) were identified from the database of the National Institute of Standards and Technology (NIST, available online: https://physics.nist.gov/PhysRefData/ASD/lines_form.html). However, these elements are not constituents of thiophanate-methyl, indicating that the elements of most interest may be shown by low-intensity emission peaks. The observed LIBS data had >18,000 variables, and many of the low-intensity peaks were indistinguishable from noise, which affected the accuracy of the analysis. Therefore, variables with low emission levels were deleted. We considered different thresholds of LIBS intensity, from 500 to 3000 at intervals of 50, for variable elimination and deleted variables with LIBS intensity less than the threshold value. The optimal threshold value was determined to be 1700 after comparing the results of partial least squares regression for the different thresholds. All further LIBS analysis was conducted using variables with intensity values >1700.

**Figure 2:**
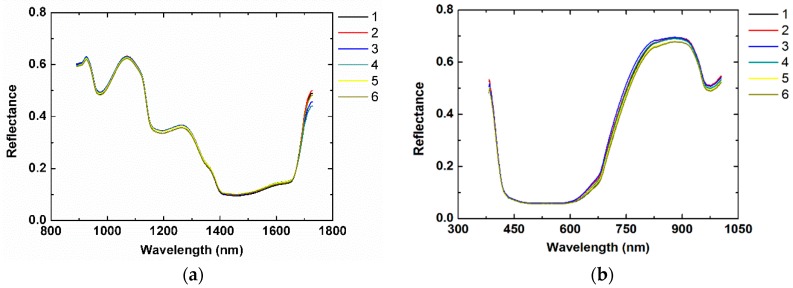
Typical Vis-NIR (visible and near infrared reflectance) hyperspectral profiles measured based on (**a**) system I and (**b**) system II for uncontaminated mulberry samples and samples contaminated with fungicide residue (concentrations of the fungicide solutions used in the samples from groups 1 to 6 were 0 g mL^−1^, 0.0050 g mL^−1^, 0.0025 g mL^−1^, 0.0017 g mL^−1^, 0.0013 g mL^−1^, and 0.0001 g mL^−1^).

The average spectral reflectance (Figure 2) in the wavelength ranges 400–1000 nm (HSI data I measured using system I) and 900–1700 nm (HSI data II measured using system II) were obtained from the region of interest (ROI) in the HSI images of the mulberry samples. Six typical spectral curves had similar trends. Five main absorption peaks in the visible and near-infrared regions were found at the 500 nm, 960 nm, 1190 nm, 1450 nm, and 1660 nm bands. The region at approximately 500 nm was assigned to the anthocyanin and chlorophyll pigments that determine the color of the fruit. The absorption peak at 960 nm was from the vibration of molecular bonds C–H, O–H, and N–H, which were from water and carbohydrates. Another two absorption peaks were associated with the C–H second stretching overtone (at 1190 nm) and the O–H first stretching overtone (at 1450 nm) in water. The region at 1661 nm was related to the C–H stretching and O–H stretching of sugar. Although the main peaks of the spectra were identified, there were no peaks directly related to thiophanate-methyl because information relevant to thiophanate-methyl was included in the overlapped spectra.

Both the LIBS and the HSI spectra do not show typical peaks that are related to thiophanate-methyl. We calculated the correlations between the thiophanate-methyl residues and each LIBS and HSI band, and the results showed that no bands of either the LIBS spectra or the HSI spectra were well correlated with the thiophanate-methyl residues. The correlations were 0.099 to 0.325 for HSI data I, −0.048 to 0.182 for HSI data II, and −0.247 to 0.261 for the LIBS data. Therefore, it is difficult to predict the thiophanate-methyl residue on the fruit by observing their spectra or depending on one absorption peak. Chemometrics was used to further qualitatively and quantitatively analyze the LIBS and HSI data.

### 2.2. PCA Analysis

Principal component analysis (PCA) was used to distinguish qualitatively between mulberry fruit samples with different thiophanate-methyl residues in order to evaluate the capacity of LIBS and HSI techniques in predicting the thiophanate-methyl residue on mulberries. PCA facilitates the interpretation of multidimensional data by reducing the dimensionality of the data; it is commonly used to analyze spectral data. PCA was used on the LIBS data, the HSI data, and a combination of the LIBS data and HSI data III (LIBS–HSI data), both before and after preprocessing the data.

**Figure 3:**
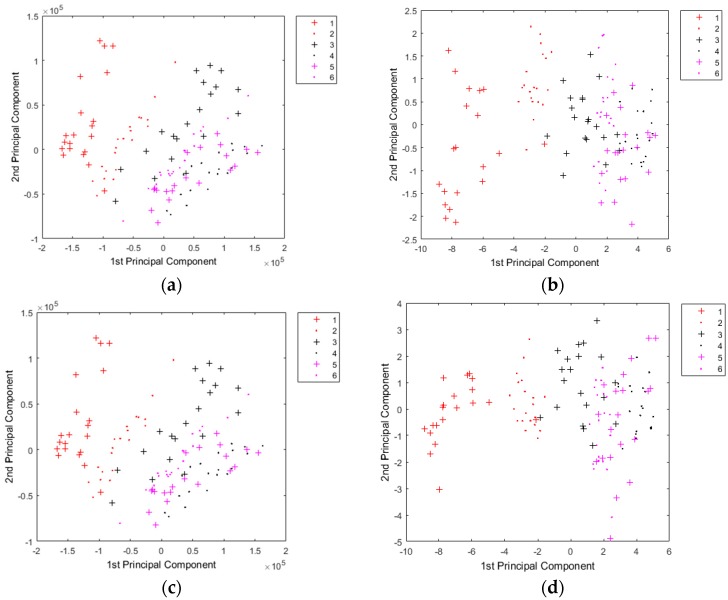
PCA plots using the LIBS data (**a**) before preprocessing and (**b**) after preprocessing, and the LIBS data combined with the HSI data (**c**) before preprocessing and (**d**) after preprocessing (concentrations of the pesticide solutions used in the samples from groups 1 to 6 were 0 g mL^−1^, 0.0050 g mL^−1^, 0.0025 g mL^−1^, 0.0017 g mL^−1^, 0.0013 g mL^−1^, and 0.0001 g mL^−1^).

Figure 3 and Supplementary Figure S1 (online) show the corresponding PCA plots based on the first two principal components. The samples with different thiophanate-methyl residues were partially separated based on the PCA plots of the LIBS data and the LIBS–HSI data (Figure 3). The contaminated fruit samples could be distinguished from the clean samples in the scatter plots of both the LIBS data and the LIBS–HSI data (Figure 3). However, it is generally difficult to distinguish between fruit samples with different thiophanate-methyl residues by directly analyzing the plots in Figure 3, unless the difference of thiophanate-methyl residues is large. For example, the samples with high thiophanate-methyl residues (1:200 and 1:400) could be distinguished from the samples with low thiophanate-methyl residues (1:600, 1:800, and 1:1000). The high residue groups could be partially separated from each other, whereas the low residue groups mostly overlap. The LIBS data and the LIBS–HSI data that were not preprocessed produced PCA plots similar to those from the preprocessed data, which indicates that the main information obtained from the LIBS data was similar to the combined spectral data from the thiophanate-methyl detection. Additionally, the cumulative contribution rates of the first two principal components of the plots in Figure 3 were >90%, indicating that most of the information from the LIBS data and from the LIBS–HSI data was explained by the first two principal components.

We also conducted PCA using the HSI data I, HSI data II, and the HSI data III before and after preprocessing (Supplementary Figure S1, online). All samples substantially overlapped and could not be accurately separated. This result means that it was difficult to distinguish mulberry fruit with or without thiophanate-methyl residue based only on the HSI data. Although the sample distributions of the PCA plots using the original data and the corresponding preprocessed data were not identical, and there is little difference between them, as shown in both Figure 3 and Supplementary Figure S1 (online). This demonstrates that preprocessing did not improve the PCA analysis in terms of distinguishing between the samples. Thus, the PCA scatter plots showed that the LIBS data was better than the HSI data for distinguishing between the different thiophanate-methyl residues of the samples.

### 2.3. Predictions Using All Variables of LIBS and HSI Data

#### 2.3.1. Analysis Using All Variables of the LIBS Data

The feasibility of using LIBS to detect thiophanate-methyl residue on mulberry fruit was investigated by creating partial least squares regression (PLSR) models to quantitatively analyze the LIBS data. Both the original data and the preprocessed data were used for model calibration to determine whether preprocessing improved model performance. The modeling results using the full set of LIBS variables are shown in Table 1.

The results indicate that the eight PLSR models (four sample sets × two forms of data, the original data and the preprocessed data) were well calibrated, with an average *R_c_* value of 0.963. The results for the independent prediction sets were also good, with an average *R_p_* value of 0.862. The best of the eight PLSR models was the model that used the preprocessed data of sample set III, which had an *R_p_* value of 0.898 and an the absolute difference between RMSEC and RMSEP (ABS) value of only 1.64 × 10^−4^. However, even the worst model had an *R_p_* value of 0.844, which shows that even when 30 different samples were selected, the PLSR model using the full set of LIBS variables gave a good prediction.

The PLSR models were used before and after data preprocessing to determine whether preprocessing improved the accuracy of the prediction. Table 1 shows that model predictions were improved when spectral data were preprocessed. Analysis of the average performance using the four sample sets showed that the models using preprocessed data had a higher average *R_c_* (0.958 vs. 0.968) and *R_p_* (0.862 vs. 0.879), and a lower average RMSEC (root mean square error of calibration) (4.49 × 10^−4^ vs. 3.74 × 10^−4^) and RMSEP (root mean square error of prediction) (8.16 × 10^−4^ vs. 7.73 × 10^−4^) than those using data that was not preprocessed. This result indicates that preprocessing increased the PLSR model accuracy.

#### 2.3.2. Analysis Using All Variables of the HSI Data

The HSI data for all variables were used to create the prediction models to determine the feasibility of using HSI to detect thiophanate-methyl residue on mulberries. Supplementary Tables S1–S3 (online) show the results for HSI data I, HSI data II, and HSI data III. In general, the predictions from HSI data I and HSI data II were worse than those from the LIBS data. Even the best model had an *R_p_* value of only 0.654, which indicates that it was difficult to detect the thiophanate-methyl residue using HSI data I or HSI data II. The eight sample sets (four original, four preprocessed) of HSI data III (HSI data I combined with HSI data II) were used to create the PLSR models. Supplementary Table S3 (online) shows the results. Prediction of HIS data III was slightly improved (the average *R_p_* value of the eight sample sets was 0.744) relative to those of HSI data I and HSI data II. However, the results from HSI data III were worse than those from the LIBS data (0.744 vs. 0.871). Preprocessing did not significantly increase prediction accuracy or the robustness of the PLSR models using HSI data.

#### 2.3.3. Analysis Using all Variables of Combined LIBS and HSI Data

We analyzed the use of combined LIBS and HSI data to determine the feasibility of detecting thiophanate-methyl residue from the combined data. The LIBS–HSI data contained 1070 variables. Table 2 shows the results of the PLSR model predictions using the combined data. Good calibration and prediction results were obtained from four sample sets before and after preprocessing. The average *R_c_* and *R_p_* values for the eight PLSR models (four sample sets × two data forms, original and preprocessed data) were 0.979 and 0.874. However, the combined LIBS and HSI data produced more accurate predictions than the LIBS data alone.

### 2.4. Prediction Using Only Optimal Variables of LIBS and HSI Data

#### 2.4.1. Analysis Using Only Optimal Variables from LIBS Data

Multidimensional LIBS data has many dimensions usually contains unrelated variables, which can affect the accuracy and robustness of the predictions. Selecting only the most important and relevant variables from the LIBS data can significantly reduce the dimensionality of the data and simplify model calibration. CARS (competitive adaptive reweighted sampling) was used to find the optimal LIBS variables. Table 1 shows the results given by the PLSR models using the optimal variables. There were on average 26 variables remaining after selection, which reduced the average number of variables by 93.26% (from 386 to 26). The average *R_c_* and *R_p_* values of the eight PLSR models after variable selection were 0.951 and 0.867. However, the predictions of the PLSR models using the optimal LIBS variables were worse than those from the full set of LIBS variables, although preprocessed data provided more accurate (average *R_p_* was 0.885 vs. 0.850) and robust (average ABS of 2.82 × 10^−4^ vs. 3.77 × 10^−4^) model performance with the sets of optimal variables.

#### 2.4.2. Analysis Using Optimal Variables from Combined LIBS and HSI Data

The optimum variables from the HSI data were not selected because the predictions from the HSI data with all variables were not adequate. Thus only optimal variables from the LIBS–HSI data were selected (1070 variables). Variables were selected, in the order of tens, from the full set of LIBS-HSI variables, and the average number of optimal variables for preprocessed data was twice the number of optimal variables for the original data (66 vs. 27). The predictions of the PLSR models were based on the optimal variables of the LIBS–HSI data. Table 2 shows the results. The average *R_p_* and RMSEP values of the eight PLSR models were 0.894 and 7.09 × 10^−4^. These results were not only better than those of the PLSR models using all LIBS–HSI variables (average *R_p_* = 0.873 and average RMSEP = 7.85 × 10^−4^), but also better than those using only the optimal LIBS variables or all the LIBS variables. The best result using the optimal LIBS–HSI variables was from preprocessed sample set II, which had the highest *R_c_* and *R_p_* values of 0.994 and 0.937, respectively; the worst result was from the original sample set IV, with *R_c_* and *R_p_* values of 0.923 and 0.837, respectively.

## 3. Discussion

We investigated the feasibility of using LIBS and HSI to detect thiophanate-methyl residues on mulberry fruit. Quantitative PLSR models were created using the LIBS and HSI data, and the results are shown in Table 1 and Table 2 and Supplementary Tables S1–S3 (online). Only the average results of the four models created from four sample sets were used, a total of fourteen models (two models/table × three tables + four models/table × two tables) were obtained. Four models, which were all derived from preprocessed data, had residual predictive deviation (RPD) values >2.0. They were respectively derived from all the LIBS variables, all the LIBS-HSI variables, the optimal LIBS variables, and the optimal LIBS–HSI variables. By considering the accuracy and robustness of the four models, the best model for the determination of thiophanate-methyl residue on the mulberry fruit was identified as the one that was derived from the preprocessed optimal LIBS–HSI variables, which had RPD = 2.585 and ABS = 4.15 × 10^−4^. These results confirm that it is feasible to use LIBS and HSI to detect thiophanate-methyl residue on mulberry fruit.

A new approach was used to detect the thiophanate-methyl residue on mulberry fruit that combined LIBS and HSI with chemometric methods and examined the results. The LIBS technique gave more accurate predictions than the HSI technique. The best model based on the LIBS data had RPD = 2.080, whereas the highest RPD of the best HSI model was only 1.485. The results show that the LIBS technique was more suitable for detecting thiophanate-methyl residue than the HSI technique. However, better results were obtained from the combination of two techniques and selecting the optimal variables.

The LIBS and the HSI data were preprocessed prior to the PCA and PLSR analyses. Analysis of the results in Table 1 and Table 2 and Supplementary Tables S1–S3 (online) showed that all five types of data in the five tables gave better predictions from preprocessed data than from the original data. The increases in RPD were 19.27% and 29.44% for the preprocessed LIBS and LIBS-HSI data, respectively, after optimal variables had been selected. The results showed the potential of standard normal variate (SNV) in increasing the accuracy of predictions from LIBS and LIBS–HSI data and the importance of preprocessing the data before analyzing it.

The observed LIBS and HSI data contained hundreds of variables. Therefore, optimal variables were selected before the subsequent PCA and PLSR analyses. The LIBS data showed no significant increase in prediction accuracy by comparing the results in Table 1 and Table 2. The average RPD values for all LIBS variables and for the optimal LIBS variables were close (2.017 vs. 1.991). However, there was a clear improvement for the LIBS–HSI data, as the average RPD value increased by 11.48% from 2.055 to 2.291 after optimal variables were selected. The ABS values show that the robustness of both the LIBS and the LIBS–HSI models improved after optimal variable selection. The average ABS value decreased by 13.84% (from 3.83 × 10^−4^ to 3.30 × 10^−4^) for the LIBS data, and the average ABS value decreased by 36.59% (from 4.81 × 10^−4^ to 3.05 × 10^−4^) for the LIBS–HSI data. Variable selection played an important role in increasing both the accuracy and the robustness of the LIBS–HSI models but did not improve the accuracy of the LIBS models.

LIBS and HSI were combined to investigate the capability of detection pesticides residues. However, this study shows two aspects of advantages and limitations of the synergic use of LIBS and HSI technologies. The main advantages of the synergic use of LIBS and HSI technologies is the combination of atomic spectroscopy (LIBS) and molecular vibrational spectroscopy (HSI). That is, the issue of pesticide residues can be analyzed from two perspectives of elemental atoms and molecular bonds. Furthermore, HSI technology can also provide the hundreds of bands of image information. Both of these merits will be beneficial for providing more analytical data for this study. On the contrary, when the LIBS and HSI technologies are combined, it brings more variables into the calibration model and leads to the increase of calculation cost during the establishment of calibration model. On the other hand, many redundant information variables are contained in the input variables and reduce the accuracy of the calibration model. Therefore, choosing two complementary technologies and appropriate variable selection methods will play an important role in establishing the accurate calibration model.

Four sample sets were used to avoid the possibility that the selection of different samples for model calibration would affect the accuracy of the PLSR models. Detailed analysis of the results given by the four sample sets showed some differences, although their results were broadly similar. The results indicated that sample selection had some influence on the model that was derived, and the use of different sample sets was important in reducing the error caused by the randomness of sample selection, increasing the accuracy of the results. Results will be more significant and more representative when there are more samples. Thus, in future studies, more mulberry samples from different varieties, habitats, climates, and years should be obtained.

The LIBS equipment detects elements and atomic and ionic emissions. Although pesticides are principally composed of complex organic molecules, some studies have used LIBS to detect pesticide contaminants in foods [18,19,20,21]. LIBS was used to detect fungicide residues on mulberry fruit, and the analysis of the LIBS data showed that detecting pesticide residue using LIBS is feasible. As shown in Figure 1, because the elements detected by LIBS were in both fruit and pesticides, the fruit samples have similar elemental compositions and no significant difference in spectral profiles between pesticide residue group and control group could be obtained via direct visual analysis. However, the unsupervised (PCA) and supervised (PLSR) models achieved good qualitative and quantitative results. This indicates that some subtle difference have been caused by pesticide residues, but they are not enough to distinguish the different groups because the LIBS emission lines of thiophanate-methyl were overlapped with that of mulberry fruit. In addition, LIBS spectra show the distribution of elements (i.e., multiple LIBS emission lines) that are associated with complex organic molecules in pesticides, and some of the substances in the fruit were changed because of the use of pesticides, resulting in a difference in the LIBS signal between the contaminated fruit and the fungicide-free fruit. All of these result in that the element from thiophanate-methyl could not be directly identified based on the LIBS emission lines. Instead, the effective chemometric methods could dig out these subtle differences (i.e., the difference in distribution of multi-emission lines in LIBS data and the difference in emission lines intensity of LIBS emission lines) between the pesticide residue group and the control group, and use these subtle differences to establish the qualitative and quantitative models. Consequently, the pesticide residue group and the control group could be effectively separated and the pesticide residues can be quantitatively detected. Although the specific reasons why LIBS can detect pesticides have not been ascertained, this study demonstrates the feasibility of using LIBS technology to detect pesticide residues. In the future, many more mulberry samples with much wider concentration ranges of thiophanate-methyl should be obtained for more accurate analysis, new variable selection methods based on the characteristics of LIBS data should be developed to further improve the detection accuracy of the model, and a rapid detection technology for pesticide that can replace HSI technology to be combined with LIBS to further improve detection accuracy should be found. Generally, more work should be done to investigate why LIBS can be used to detect thiophanate-methyl residue on mulberry fruit and how to further improve the accuracy of the detection model.

## 4. Materials and Methods

### 4.1. LIBS System and Measurement Parameters

**Figure 4:**
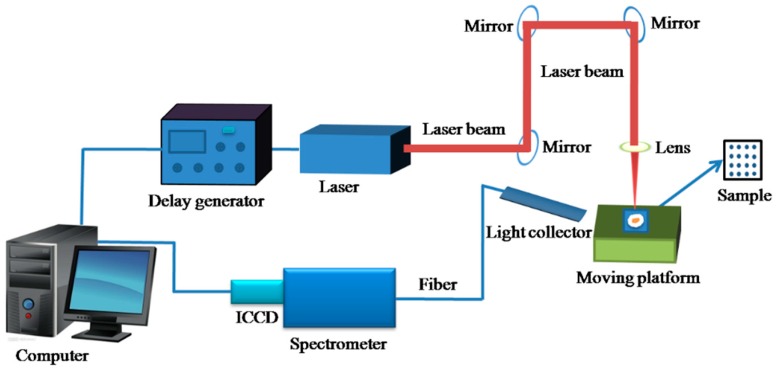
A typical LIBS analysis system setup.

Figure 4 shows a schematic of the LIBS system. The device has a Q-switched Nd:YAG pulsed laser (Vilte-200, Beamtech Optronics Co. Ltd., Beijing, China), which has a maximum energy of 200 mJ at a harmonic wavelength of 532 nm, repetition rate of 1 to 10 Hz, pulse duration of 8 ns, and beam diameter of 7 mm. The laser was used to ablate the pelletized flake samples. The LIBS device also had an echelle spectrometer (Mechelle 5000, Andor Technology, Belfast, UK) coupled with an intensified charge-coupled device (ICCD) camera (i Star DH340T-18F-03, Andor Technology, Belfast, UK). These coupled devices were used to disperse the emission spectra and collect the spectral data. A delay generator and a plano-convex lens were used to ensure the correct delay time that would eliminate the initial emission and focus the laser beam onto the surface of the sample. An X–Y–Z moving platform was used to provide the precise movement required to accurately position the sample ablation site. The computer collects and analyzes the data. The test conditions were: delay time 4 μs, integration time 16 μs, detector gain 1500, and laser pulse energy 80 mJ. The wavelengths of the spectrograph and spectral intensity were calibrated using a mercury-argon lamp (HG-1, Ocean Optical, largo, FL, USA), and a deuterium-halogen light source was used (DH-2000-BAL, Ocean Optical, USA).

### 4.2. HSI System and Measurement Parameters

Two hyperspectral imaging systems, having wavelength ranges of 400 nm to 1000 nm (system I) and 900 nm to 1700 nm (system II), were used to acquire hyperspectral images. Each system had four major components: a hyperspectral CCD camera, a moving platform, a halogen light source, and control software. Two hyperspectral CCD cameras (Spectral Imaging Ltd., Oulu, Finland) were used for spectral image acquisition: an Imspector V10E imaging spectrograph (400 nm–1000 nm) in system I and an Imspector N17E (900 nm–1700 nm) in system II. Both hyperspectral CCD cameras were the line-scanning type. The moving platform was controlled by the software to obtain a complete line-scanned image of the sample. A 150 W halogen light source provided enough light for data measurement.

### 4.3. Fruit Sample and Pesticide Preparations

The fungicide was 70% thiophanate-methyl. Its chemical name is 1,2-bis(3-(methoxycarbonyl)-2-thioureido) benzene. Thiophanate-methyl is a commonly used broad-spectrum fungicide for the treatment of plant diseases and is commonly applied by spraying on mulberry trees to prevent and treat bacterial and fungal diseases [31]. The fungicide was added to deionized water, which was then stirred to dissolve the thiophanate-methyl. Six thiophanate-methyl solutions were prepared: 0 (0 g mL^−1^), 1:200 (0.0050 g mL^−1^), 1:400 (0.0025 g mL^−1^), 1:600 (0.0017 g mL^−1^), 1:800 (0.0013 g mL^−1^), and 1:1000 (0.0001 g mL^−1^).

Fresh ripe mulberry fruit (cultivar Dashi) were harvested from a commercial mulberry field close to Huzhou city, Zhejiang province, China, and immediately transported to the laboratory. Three-hundred and sixty uniform, disease-free, unblemished, unbruised fruit were selected for our experiments. Three fruit were considered to be one sample, giving 120 samples. Fruit were exposed to the thiophanate-methyl solutions for five minutes, and then were removed and naturally air dried in a ventilated environment at room temperature for two hours.

### 4.4. LIBS and HSI Measurements

The hyperspectral images of the mulberry samples were obtained as follows. The mulberry sample was placed on the movable platform, and the light intensity and the angle of the halogen light source were adjusted. The platform was moved under the control of the computer to position the samples so that three-dimensional (x, y, λ) hyperspectral images of the fruit could be obtained. The raw images acquired by both systems I and II were then corrected to reflectance images using the method described in the literature [32]. The hyperspectral images acquired were of the intact mulberry fruit samples.

Mulberries have a high water content and a rough surface, so it is inadvisable to collect the LIBS data directly from the intact fruit. To overcome this obstacle, the fruit were dried at 65 °C for 50 h in an oven and individually powdered in a grinding machine. The powder of each fruit was pressed into a cuboid pellet at a pressure of 10 tons for 1 min. Each pellet weighed approximately 0.25 g with a length of 10 mm and thickness of 2 mm. LIBS spectral information was obtained from the prepared pellets. Each sample provided eighty spectra, which consisted of five successive shots at each of sixteen different positions (4 × 4) by automatically controlling the movement of the X–Y–Z moving platform. The system parameters for all samples were the same throughout the experiment to ensure experimental consistency.

The selection of samples for calibration and prediction could affect the performance of the models that were to be created. To minimize any selection effect, 120 samples were randomly selected and randomly divided into four equal groups of 30 samples. In each model calibration, one of the four groups of 30 samples was selected for prediction (one sample set), and the remaining three groups (90 samples) were used for calibration. After four iterations of the different groups of 30 samples being used for prediction, all 120 samples had been selected once for prediction (a total of four sample groups). The individual group and overall averages of the four models created were used to evaluate the LIBS and HSI techniques.

### 4.5. Chemometric Analysis

#### 4.5.1. Spectral Preprocessing

Preprocessing is an important step that preceded model calibration in both LIBS and HSI analysis. We used a classical data preprocessing method, standard normal variate (SNV), which is a row-oriented method, to center and scale individual spectra.

#### 4.5.2. Unsupervised Pattern Recognition

Principal component analysis (PCA) is an unsupervised algorithm that is widely used to reduce the dimensionality of multivariate data sets. The principle of PCA is to use an orthogonal transformation to convert a set of observations of possibly correlated variables into a set of values of linearly uncorrelated variables, the principal components (PCs) or principal modes of variation. After PCA, the number of PCs can be much less than the number of the original variables and the number of observations. The relative importance of the variables and their distribution can be shown graphically to indicate whether the data can be separated qualitatively.

#### 4.5.3. Model Calibration

Partial least squares regression (PLSR) is a classical method of multivariate statistical data analysis [33,34]. It mainly models the regression of multi-dependent variables on multi-independent variables. PLS is effective when there are many variables that show a high degree of internal linear correlations. The underlying principle of PLSR is to project the raw variables (X) into new latent variables (LVs).

#### 4.5.4. Variable Selection

The observed HSI and LIBS data had high dimensionality, making it necessary to use variable-selection methods to choose several optimal variables for modeling. A suitable variable-selection method can not only reduce data redundancy and remove uninformative variables but also simplify model calculations and improve model accuracy [35]. A competitive adaptive reweighted sampling (CARS) algorithm was used to select optimal variables for the HSI and LIBS data [36,37]. A more detailed introduction to CARS can be found in Li et al. [38].

#### 4.5.5. Model Evaluation

The following indicators were used to evaluate the performance of the PLSR models that were generated: root mean square error of calibration (RMSEC), root mean square error of prediction (RMSEP), correlation coefficients of calibration (*R_c_*) and prediction (*R_p_*), residual predictive deviation (RPD), and the absolute difference between RMSEC and RMSEP (ABS).

## 5. Conclusions

In this paper, the use of LIBS and HSI together with the chemometric methods PCA and PLSR were investigated to demonstrate the feasibility of the rapid detection of thiophanate-methyl residue on mulberry fruit. The results show that it is feasible to use LIBS technology for the detection and identification of thiophanate-methyl residue. Conversely, HSI technology failed to detect thiophanate-methyl residue on mulberry fruit. However, better results were obtained when the LIBS and HSI data were combined. Using CARS to select variables greatly reduced the number of variables and increased the accuracy of model predictions. The best results were provided by the PLSR model using optimal preprocessed LIBS-HSI variables; the model had an RPD value of 2.585 and an RMSEP value of 7.09 × 10^−4^. The results of this work are promising, considering that this is the first investigation into the detection of thiophanate-methyl residue on mulberry fruit by combining LIBS and HSI techniques. We have shown that there is a viable alternative to the traditional use of chemical methods that will protect consumers from eating contaminated mulberries and is thus beneficial in protecting humans from the hazards of pesticide residues.

## Figures and Tables

**Table 1:** PLSR model predictions for pesticide residue detection using LIBS data both with all variables (upper half) and with only optimal variables (lower half).

Set	Preprocessing	Number of Variables	LVs	Calibration		Prediction			ABS
				*R_c_*	RMSEC	*R_p_*	RMSEP	RPD	
I	No	386	13	0.972	3.72 × 10^−4^	0.853	8.22 × 10^−4^	1.913	4.51 × 10^−4^
II	No	386	12	0.964	4.16 × 10^−4^	0.866	8.55 × 10^−4^	1.897	4.39 × 10^−4^
III	No	386	11	0.942	5.28 × 10^−4^	0.884	7.41 × 10^−4^	2.141	2.13 × 10^−4^
IV	No	386	11	0.952	4.79 × 10^−4^	0.844	8.47 × 10^−4^	1.862	3.67 × 10^−4^
Average	No	386		0.958	4.49 × 10^−4^	0.862	8.16 × 10^−4^	1.953	3.67 × 10^−4^
I	Yes	386	12	0.979	3.24 × 10^−4^	0.854	8.20 × 10^−4^	1.919	4.96 × 10^−4^
II	Yes	386	11	0.971	3.77 × 10^−4^	0.895	7.69 × 10^−4^	2.117	3.91 × 10^−4^
III	Yes	386	9	0.935	5.59 × 10^−4^	0.898	7.23 × 10^−4^	2.265	1.64 × 10^−4^
IV	Yes	386	14	0.989	2.37 × 10^−4^	0.868	7.80 × 10^−4^	2.017	5.43 × 10^−4^
Average	Yes	386		0.968	3.74 × 10^−4^	0.879	7.73 × 10^−4^	2.080	3.98 × 10^−4^
I	No	19	13	0.937	5.51 × 10^−4^	0.848	8.57 × 10^−4^	1.848	3.06 × 10^−4^
II	No	21	18	0.948	4.99 × 10^−4^	0.849	9.63 × 10^−4^	1.638	4.64 × 10^−4^
III	No	36	8	0.928	5.88 × 10^−4^	0.869	7.95 × 10^−4^	1.992	2.07 × 10^−4^
IV	No	26	15	0.975	3.49 × 10^−4^	0.832	8.81 × 10^−4^	1.786	5.32 × 10^−4^
Average	No	26		0.947	4.97 × 10^−4^	0.850	8.74 × 10^−4^	1.816	3.77 × 10^−4^
I	Yes	24	8	0.958	4.52 × 10^−4^	0.873	8.11 × 10^−4^	2.053	3.59 × 10^−4^
II	Yes	36	9	0.960	4.39 × 10^−4^	0.915	6.56 × 10^−4^	2.400	2.17 × 10^−4^
III	Yes	24	7	0.940	5.37 × 10^−4^	0.907	6.71 × 10^−4^	2.347	1.34 × 10^−4^
IV	Yes	19	12	0.959	4.48 × 10^−4^	0.844	8.65 × 10^−4^	1.866	4.17 × 10^−4^
Average	Yes	26		0.954	4.69 × 10^−4^	0.885	7.51 × 10^−4^	2.166	2.82 × 10^−4^

LVs: latent variables; *R_c_*: correlation coefficients of calibration; RMSEC: root mean square error of calibration; *Rp*: correlation coefficients of calibration–prediction; RMSEP: root mean square error of prediction; RPD: residual predictive deviation; ABS: the absolute difference between RMSEC and RMSEP.

**Table 2:** Pesticide residue predictions given by the PLSR models using combined LIBS and HSI data, both with all variables (upper half) and with only optimal variables (lower half).

Set	Preprocessing	Number of Variables	LVs	Calibration		Prediction			ABS
				R_c_	RMSEC	R_p_	RMSEP	RPD	
I	No	1070	13	0.972	3.72 × 10^−4^	0.853	8.22 × 10^−4^	1.913	4.51 × 10^−4^
II	No	1070	12	0.964	4.16 × 10^−4^	0.866	8.55 × 10^−4^	1.897	4.39 × 10^−4^
III	No	1070	11	0.942	5.28 × 10^−4^	0.884	7.41 × 10^−4^	2.141	2.13 × 10^−4^
IV	No	1070	14	0.980	3.16 × 10^−4^	0.852	8.38 × 10^−4^	1.890	5.22 × 10^−4^
Average	No	1070		0.964	4.08 × 10^−4^	0.864	8.14 × 10^−4^	1.960	4.06 × 10^−4^
I	Yes	1070	16	0.987	2.52 × 10^−4^	0.883	7.40 × 10^−4^	2.125	4.88 × 10^−4^
II	Yes	1070	18	0.994	1.75 × 10^−4^	0.921	6.38 × 10^−4^	2.573	4.63 × 10^−4^
III	Yes	1070	18	0.992	1.98 × 10^−4^	0.906	7.41 × 10^−4^	2.162	5.42 × 10^−4^
IV	Yes	1070	17	0.994	1.75 × 10^−4^	0.82	9.07 × 10^−4^	1.736	7.32 × 10^−4^
Average	Yes	1070		0.992	2.00 × 10^−4^	0.883	7.57 × 10^−4^	2.149	5.56 × 10^−4^
I	No	26	15	0.944	5.18 × 10^−4^	0.866	7.99 × 10^−4^	1.971	2.81 × 10^−4^
II	No	26	7	0.916	6.31 × 10^−4^	0.881	7.52 × 10^−4^	2.093	1.21 × 10^−4^
III	No	23	7	0.916	6.29 × 10^−4^	0.880	7.53 × 10^−4^	2.096	1.23 × 10^−4^
IV	No	34	7	0.923	6.06 × 10^−4^	0.837	8.61 × 10^−4^	1.827	2.55 × 10^−4^
Average	No	27		0.925	5.96 × 10^−4^	0.866	7.91 × 10^−4^	1.997	1.95 × 10^−4^
I	Yes	72	12	0.993	1.84 × 10^−4^	0.935	5.83 × 10^−4^	2.754	3.99 × 10^−4^
II	Yes	72	14	0.994	1.67 × 10^−4^	0.937	5.67 × 10^−4^	2.852	4.00 × 10^−4^
III	Yes	56	12	0.986	2.59 × 10^−4^	0.932	6.01 × 10^−4^	2.620	3.42 × 10^−4^
IV	Yes	64	9	0.988	2.38 × 10^−4^	0.881	7.57 × 10^−4^	2.116	5.19 × 10^−4^
Average	Yes	66		0.991	2.12 × 10^−4^	0.921	6.27 × 10^−4^	2.585	4.15 × 10^−4^

## Supplementary Materials

Supplementary materials can be found at https://www.mdpi.com/1422-0067/20/8/2017/s1.

## Abbreviations

LIBS	Laser induced breakdown spectroscopy
HSI	Hyperspectral imaging
PLSR	Partial least squares regression
GC	Gas chromatography
GC-MS	Gas chromatography–mass spectrometry
HPLC	Liquid chromatography
UPLC-MS	Ultra-high-performance liquid chromatography–mass spectrometry
ICCD	Intensified charge-coupled device
SNV	Standard normal variate
PCA	Principal component analysis
PC	Principal component(s)
LVs	Latent variables
CARS	Competitive adaptive reweighted sampling
RMSEC	Root mean square error of calibration
RMSEP	Root mean square error of prediction
R_c_	Correlation coefficients of calibration
R_p_	Correlation coefficients of calibration–prediction
RPD	Residual predictive deviation
ABS	The absolute difference between RMSEC and RMSEP
NIST	National Institute of Standards and Technology
ROI	Region of interest
