# Supplementary material for: Feasibility of Laser-Induced Breakdown Spectroscopy and Hyperspectral Imaging for Rapid Detection of Thiophanate-Methyl Residue on Mulberry Fruit
Source: Int J Mol Sci. 2019 Apr 24;20(8):2017. doi: 10.3390/ijms20082017 (PMC6515382; doi:10.3390/ijms20082017)
Supplement: Supplementary file 1 [file ijms-20-02017-s001.pdf]

Feasibility of Laser-Induced Breakdown Spectroscopy and Hyperspectral Imaging for Rapid Detection of Thiophanate-Methyl Residue on Mulberry Fruit

Di Wu [1,†], Liuwei Meng [2,†], Liang Yang [2], Jingyu Wang [2], Xiaping Fu [3], Xiaoqiang Du [3,4], Shaojia Li [1], Yong He [5] and Lingxia Huang [2,6,*]

[1] College of Agriculture & Biotechnology/Zhejiang Provincial Key Laboratory of Horticultural Plant Integrative Biology/The State Agriculture Ministry Laboratory of Horticultural Plant Growth, Development and Quality Improvement, Zhejiang University, Zijingang Campus, Hangzhou 310058, China; di_wu@zju.edu.cn (D.W.); 11216044@zju.edu.cn (S.L.).

[2] College of Animal Sciences, Zhejiang University, Hangzhou 310058, China; 21517069@zju.edu.cn (M.W.); lyoung1101@163.com (L.Y.); Jingyu529@zju.edu.cn (J.W.)

[3] Faculty of Mechanical Engineering & Automation, Zhejiang Sci-Tech University, Hangzhou 310018, China; fuxp@zstu.edu.cn (X.F.); xqiangdu@zstu.edu.cn (X.D.).

[4] Key Laboratory of Transplanting Equipment and Technology of Zhejiang Province, Hangzhou, 310018, China

[5] College of Biosystems Engineering and Food Science, Zhejiang University, Zijingang Campus, Hangzhou 310058, China; yhe@zju.edu.cn

[6] South Taihu Agricultural Technology Extension Center in Huzhou, Zhejiang University, Huzhou 313000, China

* Correspondence: lxhuang@zju.edu.cn; Tel.: +86-571-88982459

† These authors contributed equally to this work.

Supplemental Figure A1. PCA plots using the data of HSI I (**a**) before preprocessing and (**b**) after preprocessing, HSI II (**c**) before preprocessing and (**d**) after preprocessing and HSI data III (**e**) before preprocessing and (**f**) after preprocessing (concentrations of the pesticide solutions used in the samples from groups 1 to 6 were 0 g mL^{-1}, 0.0050 g mL^{-1}, 0.0025 g mL^{-1}, 0.0017 g mL^{-1}, 0.0013 g mL^{-1} and 0.0001 g mL^{-1}) .

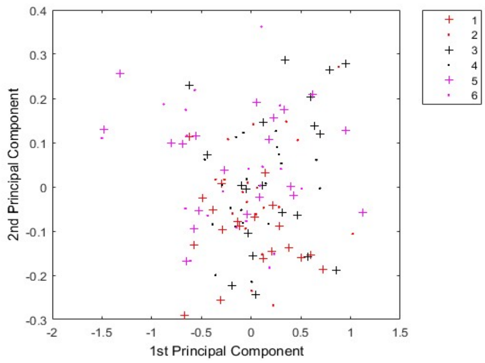

(a)

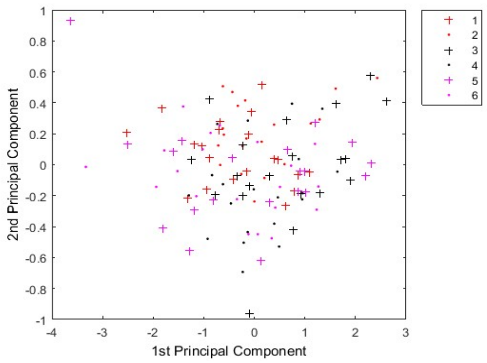

(b)

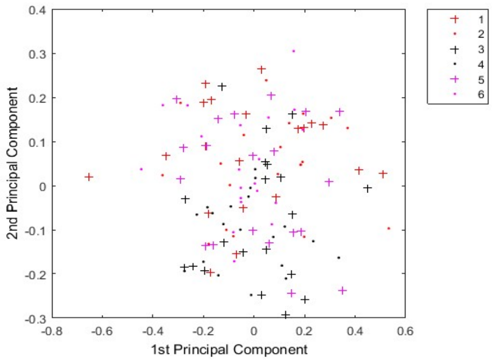

(c)

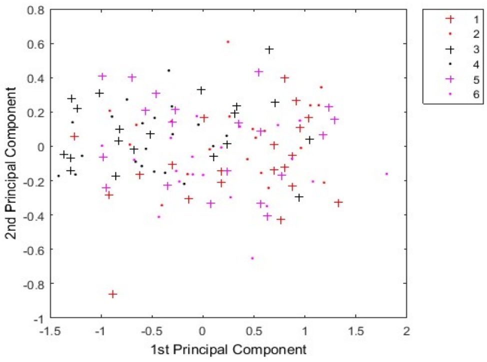

(d)

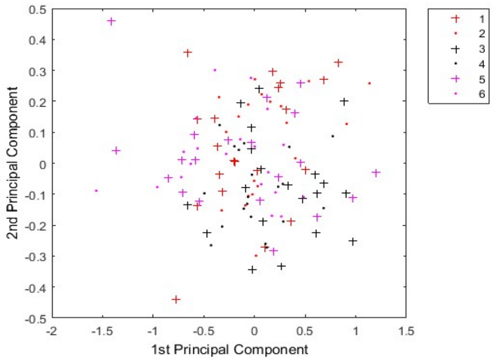

(e)

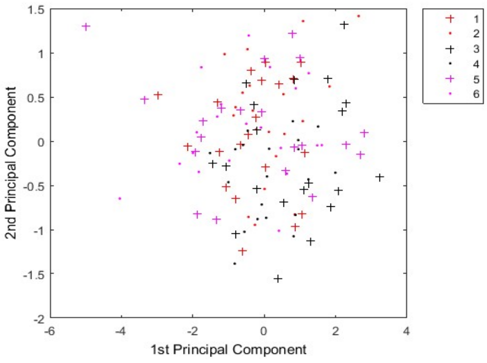

(f)

Supplementary Table A1

PLSR models predictions for pesticide residue detection using HSI data I (400 nm–1000 nm) with all variables

Set	Preprocessing	LVs	Calibration		Prediction			ABS
			R_c	RMSEC	R_p	RMSEP	RPD	
I	No	7	0.605	1.25×10^{-3}	0.403	1.46×10^{-3}	1.076	2.09×10^{-4}
II	No	12	0.756	1.03×10^{-3}	0.654	1.22×10^{-3}	1.321	1.86×10^{-4}
III	No	10	0.678	1.16×10^{-3}	0.462	1.46×10^{-3}	1.083	3.04×10^{-4}
IV	No	8	0.56	1.30×10^{-3}	0.604	1.26×10^{-3}	1.253	$4.30\times10^{-5}5$
Average	No		0.65	1.19×10^{-3}	0.531	1.35×10^{-3}	1.183	1.85×10^{-4}
I	Yes	6	0.599	1.26×10^{-3}	0.399	1.46×10^{-3}	1.076	2.02×10^{-4}
II	Yes	11	0.776	9.92×10^{-4}	0.616	1.29×10^{-3}	1.259	2.95×10^{-4}
III	Yes	11	0.790	9.65×10^{-4}	0.600	1.34×10^{-3}	1.195	3.74×10^{-4}
IV	Yes	6	0.513	1.35×10^{-3}	0.611	1.25×10^{-3}	1.260	1.01×10^{-4}
Average	Yes		0.670	1.14×10^{-3}	0.557	1.33×10^{-3}	1.198	2.43×10^{-4}

Supplementary Table A2

PLSR models predictions for pesticide residue detection using HSI data II (900 nm–1700 nm) with all variables

Set	Preprocessing	LVs	Calibration		Prediction			ABS
			R_c	RMSEC	R_p	RMSEP	RPD	
I	No	8	0.580	1.28×10^{-3}	0.374	1.51×10^{-3}	1.041	2.31×10^{-4}
II	No	7	0.545	1.32×10^{-3}	0.431	1.42×10^{-3}	1.108	1.00×10^{-4}
III	No	2	0.308	1.50×10^{-3}	0.317	1.50×10^{-3}	1.054	1.33×10^{-7}
IV	No	5	0.433	1.42×10^{-3}	0.428	1.43×10^{-3}	1.106	1.34×10^{-5}
Average	No		0.466	1.38×10^{-3}	0.388	1.47×10^{-3}	1.077	8.61×10^{-5}
I	Yes	3	0.431	1.42×10^{-3}	0.312	1.52×10^{-3}	1.035	1.01×10^{-4}
II	Yes	5	0.524	1.34×10^{-3}	0.396	1.45×10^{-3}	1.089	1.05×10^{-4}
III	Yes	4	0.503	1.36×10^{-3}	0.398	1.46×10^{-3}	1.075	1.04×10^{-4}
IV	Yes	3	0.364	1.47×10^{-3}	0.519	1.38×10^{-3}	1.143	8.22×10^{-5}
Average	Yes		0.456	1.40×10^{-3}	0.406	1.45×10^{-3}	1.086	9.82×10^{-5}

Supplementary Table A3

PLSR models predictions for pesticide residue detection using HSI data III (the combination of HSI data I and II) with all variables

Set	Preprocessing	LVs	Calibration		Prediction			ABS
			R_c	RMSEC	R_p	RMSEP	RPD	
I	No	17	0.935	5.59×10^{-4}	0.665	1.19×10^{-3}	1.338	6.29×10^{-4}
II	No	19	0.962	4.32×10^{-4}	0.712	1.14×10^{-3}	1.383	7.06×10^{-4}
III	No	13	0.842	8.49×10^{-4}	0.759	1.03×10^{-3}	1.535	1.82×10^{-4}
IV	No	15	0.886	7.31×10^{-4}	0.773	1.02×10^{-3}	1.544	2.89×10^{-4}
Average	No		0.906	6.43×10^{-4}	0.727	1.09×10^{-3}	1.450	4.52×10^{-4}
I	Yes	20	0.969	3.88×10^{-4}	0.725	1.13×10^{-3}	1.451	7.39×10^{-4}
II	Yes	17	0.945	5.15×10^{-4}	0.708	1.14×10^{-3}	1.377	6.28×10^{-4}
III	Yes	20	0.977	3.39×10^{-4}	0.817	1.05×10^{-3}	1.54	7.08×10^{-4}
IV	Yes	17	0.938	5.47×10^{-4}	0.791	1.00×10^{-3}	1.574	4.52×10^{-4}
Average	Yes		0.957	4.47×10^{-4}	0.760	1.08×10^{-3}	1.485	6.32×10^{-4}
